# Reductive activity of free and immobilized cells of cyanobacteria toward oxophosphonates—comparative study

**DOI:** 10.1007/s10811-016-0952-y

**Published:** 2016-09-15

**Authors:** Monika Górak, Ewa Żymańczyk-Duda

**Affiliations:** Department of Bioorganic Chemistry, Wrocław University of Technology, Wybrzeże Wyspiańskiego 27, 50-370 Wrocław, Poland

**Keywords:** Biotransformation, Cyanobacteria, Immobilization, Phosphonates

## Abstract

This report, based on the previous studies, compares the reductive activity of different modes of following photobiocatalysts (on laboratory and preparative scale): *Arthrospira maxima*, *Nostoc* cf. *muscorum* and *Nodularia sphaerocarpa*, toward diethyl esters of 2-oxopropylphosphonate (1), 2-oxo-2-phenylethylphosphonate (2), and 2-oxobutylphosphonate (3). It was confirmed that immobilization in alginate matrix do not affect the activity and viability of the biocatalysts. Corresponding (*S*)-hydroxyphosphonates (1a-3a) were obtained with similar efficiency compared to the free-cell mode with the yield and of the optical purity *e.e* respectively (e.g., *N. sphaerocarpa* experiments): (1) yield: 21 %, *e.e*. 84 %; (2) yield 97 %, *e.e*. 97; (3) yield 21 %, *e.e.* 89 %. Scaling up the processes for the best biocatalyst, *N. sphaerocarpa*, indicated that the use of free-living cells of cyanobacteria is more effective (640 mg of substrate 2, 44 % of yield, 91 % of *e*.*e*.), compared to the column bioreactor packed with immobilized cells of this photobiocatalyst (384 mg of substrate 2, 38 % of yield, 86 % of *e.e*). In the case of free and immobilized cells of *N.* cf. *muscorum*, agitation of the medium was the crucial activity mediator. Shaking culture of free cells of *N.* cf. *muscorum* converted the diethyl 2-oxo-2-phenylethylphosphonate (2) with the yield of 43 % (99 % of *e.e.*) compared to 18 % (99 % of *e.e.*, stationary culture). Immobilized cells of this cyanobacterium were also more active toward (2) under shaking conditions (28 % of yield, 99 % of *e.e.*) than free ones without agitation.

## Introduction

Biocatalytic processes represent an effective and, in some cases, preferable alternative to the chemical synthesis of optically pure chiral fine chemicals. The remarkable advantages of biocatalysis are its selectivity and usual lack of side products (Faber 2011). The most significant drawback of bioconversion is the requirement of aqueous environment for most enzymatic reactions, which is a restriction for biotransformation of poorly soluble organic compounds.

Current applications of biocatalyzed processes are targeted to multiple industrial recipients such as producers of detergents, pharmaceuticals, fine chemicals, food, biofuels, or animal feed (Adrio and Demain 2014). The practicability of technological solutions based upon the enzymatic systems is often limited by the problems with the loss of catalytic activity, which is often a consequence of non-physiological process conditions or/and of the toxic impact of the xenobiotic substrate. These are solved, at least partially, by biocatalyst and reaction environment engineering. The cell or enzyme immobilization techniques protect and stabilize the fragile biological structures. As a consequence, the use of free cells in industrial processes is rather rare in comparison to immobilized cells, since it offers various advantages and financial benefits (Sayed et al. 2007).

Recently, cyanobacteria have gained attention because of their applications to bioconversions of structurally different substrates into desired, usable products. In general, most of immobilizations methods used for microorganisms can be introduced into cyanobacterial biotransformations. Several techniques of immobilization of these microbes have been reported in the literature (Moreno-Garrido 2008), and usually, the specific procedure is matched to a defined biotechnological application. The entrapment of living cells in natural polysaccharide gels (calcium alginate, agar, carrageenan) or inside synthetic polymers (acrylamide, urethane, photo-crosslinkable resin) (Rooke et al. 2008; Ueno et al. 2008; Arabi et al. 2010) are the most frequently applied methods. Immobilized algae are mainly used for wastewater treatment (Lee et al. 2013), for removal of heavy metals (biosorption), e.g., the AlgaSORB^©^ sorption process uses algal cells immobilized in a silica gel polymer to remove heavy metal ions from aqueous solutions (Prasad et al. 2006). Entrapped cells of microalgae also have been used as biocatalyst in biotransformations of monoterpenes and steroids (Arabi et al. 2010; Rasoul-Amini et al. 2011). Immobilization efficiently protects cyanobacteria against direct contact with the organic solvent (Perullini et al. 2014). Such a form of biocatalyst is used in culture collection handling. This method is cheaper and a more readily available approach in the cultivation of microalgae (Gaudin et al. 2006). Cyanobacteria possess unique and interesting features among the photosynthetic microorganisms applied for energy conversion applications. Some efforts have also been made to produce electricity, using the cyanobacterium *Nostoc* sp. as a photobiocatalyst immobilized on carbon nanotubes on the anode of photobioelectrochemical cells (Sekar et al. 2014).

This paper is focused on the application of the immobilized cells of cyanobacteria as biocatalyst for a preparative scale reduction of β-oxoalkylphosphonates. Different from other possible solutions, cyanobacteria represent a diverse and unusual catalytic ability to transform β-oxoalkylphosphonates. Previously described biotransformations of β-oxophosphonates present the effective use of free cells of photoautotrophic microorganisms as whole-cell biocatalysts for the synthesis of chiral (*S*)-2-hydroxyalkylphosphonates (Górak and Żymańczyk-Duda 2015). Hydroxyphosphonates are a class of organophosphorus compounds with possible biological activity—they represent a synthetic platform for further applications (e.g., aminophosphonic acids and their derivatives) (Yokomatsu et al. 2003; Kolodiazhnyi 2006). The species, *Nodularia sphaerocarpa*, showed diverse and unusual biocatalytic ability to reduce the β-oxophosphonates, which manifests, among others, as the capacity to reduce substrate toward which other biocatalysts were non-active (Żymańczyk-Duda et al. 2000; Żurawiński et al. 2001). However, these processes allowed product formation only as a result of bioconversions carried out in closed vessels without the possibility of feeding and reusing them. The inhibition properties of phosphonate derivatives (toward many enzymes) as well as scaling up the bioreactions and biocatalysts reusing are the restrictive steps. To overcome these, the cells of biocatalysts, *Arthrospira maxima*, *Nostoc* cf. *muscorum*, and *N. sphaerocarpa*, were immobilized by entrapment method, using sodium alginate as supporting material.

This paper reports a new approach, which allowed the production of optically pure diethyl β-hydroxyphosphonates on the larger scale. The aim of this study was to invent the effective immobilization method for the oxo-xenobiotics*—*β-oxoalkylphosphonates enantioselective reduction. Thus, several efforts have been undertaken to optimize the entrapment process to obtain a stable culture of immobilized cells as well as a stable structure of alginate beads for bioreactor applications. To confirm the possibility of application of free-living and immobilized cells of *N. sphaerocarpa* in the preparative scale batch culture and a continuous-flow photobioreactor, appropriate experiments were conducted.

## Materials and methods

### Chemicals

All chemicals were purchased from commercial suppliers: Sigma, Aldrich, Fluka, Avantor Performance Materials Poland S.A.

### Synthesis of substrates

The substrates, diethyl 2-oxopropylphosphonate (96 %; obtained from chloroacetone), diethyl 2-oxo-2-phenylethylphosphonate (97 %; obtained from 2-bromoacetophenone), diethyl 2-oxobutylphosphonates (96 %; obtained from 1-bromo-2-butanone), were synthesized according to standard procedure described in literature (Ryglowski and Kafarski 1996).

Chloroacetone (54 mmol) was mixed with saturated aqueous solution of sodium iodide (54 mmol) and methanol. The mixture was stirred for 24 h at room temperature, methanol was evaporated under reduced pressure, and 20 mL of water was added. The iodoketone was extracted with two portions of chloroform (2 × 30 mL). The combined extracts were dried over magnesium sulfate. To iodoacetone (or 2-bromoacetophenone or 1-bromo-2-butanone) equimolar quantity of triethyl phosphite were added dropwise maintaining temperature at 5 °C. The mixture was left overnight at room temperature and the volatile components of the reaction were removed on a rotary evaporator. The crude product was purified by means of column chromatography on silica gel using ethyl acetate as eluent.

### Microorganisms

Axenic strains of *Arthrospira maxima* (CCALA 27*)*, *Nodularia sphaerocarpa* (CCALA 114), and *Nostoc* cf. *muscorum* (CCALA 129) were from the Culture Collection of Autotrophic Organisms (CCALA) of the Institute of Botany of the Academy of Sciences of the Czech Republic.

### Culture conditions

All species were cultivated in 250-mL Erlenmeyer flasks containing 100 mL of a suitable medium. *Nodularia sphaerocarpa* was grown on BG-11 medium (pH 7.0) (Rippka et al. 1979), *N.* cf. *muscorum* was grown on BG-11 medium without NaNO_3_ (pH 7.0), and *A. maxima* on *Spirulina* medium (pH 9.5) (Aiba and Ogawa 1977). All species of cyanobacteria were cultivated under continuous illumination at 7–12 μmol photons m^−2^ s^−1^ (Power Glo fluorescent bulb, 8 W, Hagen) at 28 °C (±1) under stationary conditions. Power Glo fluorescent bulb illuminates with bluish white light. The experiments were conducted under sterile conditions.

### Cell immobilization by gel entrapment

The cyanobacteria entrapped in alginate beads were prepared using 21-day-old cultures of cyanobacterial strains. Cells were harvested by centrifugation at 1788×*g* at 17 °C for 20 min. Then, the cells of *N. sphaerocarpa* and *A. maxima* were suspended in 5 mL of BG-11 medium and distilled water, respectively, whereas the cells of *N.* cf. *muscorum* were suspended in 10-mL BG-11 medium without NaNO_3_. Cyanobacterial cells were mixed homogenously with equal volume of a sodium alginate solution (2 or 4 %, *w*/*v*) for 20 min, to obtain a final concentration of 1 and 2 % (*w*/*v*) of alginate/cells mixture. Then, the mixture was dropped into cold solution of CaCl_2_·2H_2_O (0.2 M) by syringe, forming gel beads with diameters between 2.0 and 3.0 mm. Pump pressure and a needle gauge were used to control the bead size. The beads were incubated at room temperature for 1 h. Then, the alginate beads were washed with distilled water, placed in BaCl_2_·2H_2_O solution (0.85 %) for 15 min, rinsed again with distilled water and then with medium suitable for tested cyanobacterial strain. The produced Ca-alginate beads were cultured in fresh and sterile medium. Immobilized cells of *N. sphaerocarpa* and ***N.*** cf. *muscorum* were cultivated on BG-11 and BG-11 without NaNO_3_, respectively. Alginate-entrapped cells of *A. maxima* were suspended in sterile, distilled water.

### General procedure of *β*-oxoalkylphosphonates bioreduction

#### Immobilized cells

After immobilization, the alginate-entrapped cells were incubated in 100-mL Erlenmeyer flasks containing 50 mL of a suitable medium and 2 mM of the substrate (20 mg of compounds 1 and 3 and 26 mg of compound 2).

#### Free-living cells

The cyanobacteria were cultivated under continuous illumination (Power Glo lamp) at 31 °C (±1) under shaking (70 rpm) conditions. After 21 days of precultivation, 1 mM of substrate (20 mg of compounds 1 and 3 and 26 mg of compound 2) was added to the culture of cyanobacteria (100 mL).

The bioconversions were carried out for 7 days, at 31 °C (±1), under shaking (70 rpm) conditions and under continuous bluish white light (Power Glo). Experiments were completed by the biomass removing by filtration—in the case of immobilized cells or by centrifugation (2800×*g*, 25 min, 17 °C) of free cells of cyanobacteria. Finally, the supernatants were extracted twice with 50 mL of ethyl acetate. Subsequently, collected organic layers were dried over anhydrous MgSO_4_ and evaporated under reduced pressure. The resulting oils were analyzed.

The control experiments were carried out in culture medium without as well as the biocatalysts and the substrate.

The conversion degrees of the substrates and the optical purity of the products were evaluated using the NMR technique. Each experiment was replicated in parallel at least three times.

### Preparative biotransformations of diethyl 2-oxo-2-phenylethylphosphonate (2)

#### Immobilized cells

For continuous flow column experiments, a preparative scale glass bioreactor of 40-cm length and 2-cm internal diameter was used. The bioreactor was packed with immobilized cells of *N. sphaerocarpa* (33 cm height). Biomass obtained from ten cultures of *N. sphaerocarpa* (10 × 100 mL) was immobilized by gel entrapment (2 % (*w*/*v*) of sodium alginate). The BG-11 medium (150 mL) with 10 mM (384 mg) of diethyl 2-oxo-2-phenylethylphosphonate (2) was pumped from a reservoir into the reactor at 4 mL min^-1^ flow rates with the help of a peristaltic pump. The bioprocess was conducted for 5 days under photoperiod of 16 h/8 h (day/night) provided by natural daylight intensity at 27 °C (±1) in growth room. Experiments were carried out in June and July 2014 (Wrocław, 51° 06′ 36″ N, 17° 01′ 20″ E). At regular intervals (24 h), eluents were collected and the presence of diethyl 2-hydroxy-2-phenylethylphosphonate (2a) in these fractions was detected.

#### Free-living cells

*Nodularia sphaerocarpa* was cultivated in 2000-mL Erlenmeyer flasks containing 500-mL BG-11 medium, under continuous illumination provided by Power Glo fluorescent bulb, at 31 °C (±1) under shaking conditions (70 rpm). After 21 days of precultivation, 5 mM (640 mg) of compound 2 was added to the culture. The experiments were completed after 7 days.

### Optical purity assignment

The mixtures of bioconversion products were analyzed by ^31^P NMR spectroscopy. Spectra were recorded on Bruker Avance 600 instrument operating at 600 MHz, and measurements were made in CDCl_3_ (99.5 at % D) at temperature of 298 K.

The optical purity of the products was estimated using quinine as a chiral solvating agent, which allowed achieving a shift difference of ^31^P NMR signals coming from the hydroxyphosphonate–bioreduction products (Żymańczyk-Duda et al. 1996).

The degree of the conversion of the substrate was expressed as a percentage (%) and defined as

$$ \mathrm{conversion}=\frac{PP}{PP+PS}\times 100\%, $$

where *PP* and *PS* are the areas under the signals observed on the ^31^P NMR spectrum, coming from product and substrate of bioconversion, respectively.

Optical purity was computed also from the ^31^P NMR spectrum following the formula:

$$ e.e.=\frac{E1-E2}{E1+E2}\times 100\%, $$

where *E1* and *E2* are the values of the area under the signals coming from the major and minor enantiomer of the product, respectively.

### Spectroscopic data

#### Diethyl 2-oxopropylphosphonate (1)

^31^P NMR (600 Hz, CDCl_3_) δ 19.74; ^1^H NMR (600 Hz, CDCl_3_) δ 1.34 (t, 6 H, *J* = 7.1 Hz, P(O)(OCH_2_C*H*_3_)), 2.33 (s, 3 H, C*H*_3_C(O)), 3.1 (d, 2 H, *J* = 22.9 C*H*_2_P), 4.1–4.2 (m, 4 H, m, P(O)(OC*H*_2_CH_3_)).

#### Diethyl 2-hydroxypropylphosphonate (1a)

^31^P NMR (600 Hz, CDCl_3_) δ 30.11; ^1^H NMR (600 Hz, CDCl_3_) δ 1.30 (dd, 3 H, *J* = 6.1, *J* = 2.2, C*H*_3_C(OH)), 1.35 (t, 6 H, *J* = 7.1 Hz, P(O)(OCH_2_C*H*_3_)), 1.9–1.98 (m, 2 H, C*H*_2_P), 3.55 (s, 1 H, C(O*H*)), 4.08–4.25 (m, 4 H, P(O)(OC*H*_2_CH_3_)).

#### Diethyl 2-oxo-2-phenylethylphosphonate (2)

^31^P NMR (600 Hz, CDCl_3_) δ 20.58; ^1^H NMR (600 Hz, CDCl_3_) δ 1.29 (t, 6 H, *J* = 7.0 Hz, P(O)(OCH_2_C*H*_3_)), 3.65 (d, 2 H, *J* = 22.7, C*H*_2_P), 4.15 (m, 4 H, P(O)(OC*H*_2_CH_3_)), 7.3–7.6 (m, 5 H, C_6_H_5_).

#### Diethyl 2-hydroxy-2-phenylethylphosphonate (2a)

^31^P NMR (600 Hz, CDCl_3_) δ 29.76;

^1^H NMR (600 Hz, CDCl_3_) δ 1.29 (t, 6 H, *J* = 7.1, P(O)(OCH_2_C*H*_3_)), 2.14–2.28 (m, 2 H, C*H*_2_P), 4.05–4.20 (m, 4H P(O)(OC*H*_2_CH_3_)), 5.07–5.14 (m, 1H, C(OH)*H*), 7.25–7.43 (m, 5H, C_6_H_5_).

#### Diethyl 2-oxobutylphosphonate (3)

^31^P NMR (600 Hz, CDCl_3_) δ 20.08; ^1^H NMR (600 Hz, CDCl_3_) δ 1.04 (t, 3 H, *J =* 7.2 Hz, C*H*_3_CH_2_C(O)), 1.31 (t, 6 H, *J* = 7.0 Hz, P(O)(OCH_2_C*H*_3_)), 2.65 (q, 2 H, *J* = 7.2 Hz, CH_3_C*H*_2_C(O)), 3.06 (d, 2 H, C*H*_2_P, *J* = 22.8 Hz), 4.05–4.19 (m, 4 H, P(O)(OC*H*_2_CH_3_)).

#### Diethyl 2-hydroxybutylphosphonate (3a)

^31^P NMR (600 Hz, CDCl_3_) δ 30.81; ^1^H NMR (600 Hz, CDCl_3_) δ 0.95 (t, 3 H, *J =* 7.2 Hz, C*H*_3_CH_2_C(O)), 1.33 (t, 6 H, *J* = 7.0 Hz, P(O)(OCH_2_C*H*_3_)), 1.49–1.62 (m, 2 H, CH_3_C*H*_2_CH(OH)), 1.82–2.00 (m, 2 H, C*H*_2_P), 2.22 (s, 1 H, C*H*(OH)), 3.56 (s, 1 H, CH(O*H*)), 4.06–4.18 (m, 4 H, P(O)(OC*H*_2_CH_3_)).

### Purification of diethyl 2-hydroxyphosphonates (1a, 2a, 3a)

Diethyl 2-hydroxyphosphonates were purified by reversed-phase pressure liquid chromatography (C18—reversed phase silica gel, partial size 15–25 μm, pore size 100 Å) using a mixture of water–acetonitrile (10:2) as an eluent, flow rate of 3 mL min^−1^.

### Determination of absolute configuration of diethyl 2-hydroxyphosphonates (1a, 2a, 3a)

Absolute configuration of diethyl 2-hydroxyphosphonates (1a, 2a, 3a) were determined according to the procedure previously described (Górak and Żymańczyk-Duda 2015).

## Results

### Immobilized cells vs. free cells of cyanobacteria

This paper presents the comparative study of reductive activity of immobilized and free cells of cyanobacteria. To overcome the inhibition properties of oxophosphonates toward enzymatic systems of the cells, biocatalysts were immobilized. The concentration of the applied solutions of sodium alginate was 2 and 4 % and the final concentrations of alginate in the prepared beads were 1 and 2 %, respectively. First of all, microalgal beads containing a final concentration of 2 % (*w*/*v*) of alginate were used as biocatalysts, but in this case, a decrease of conversion of substrates compared to the process with free cells, cultivated under shaking (70 rpm) condition, was observed (Table 1). It is possible that high concentration of alginate caused limitations in the substrate—immobilized biocatalyst contact. The application of 1 % (*w*/*v*) of alginate for bead preparation did not affect the cell viability, and this solution was slightly more effective compared to the activity of the cells entrapped in the matrix made of 2 % alginate (except from *N. sphaerocarpa* and substrate 1) (Table 1). Experiments with beads containing ultimately 0.5 % (*w*/*v*) of alginate were not performed, because even 1 % (*w*/*v*) of initial concentration of sodium alginate resulted in weak beads, and in some cases, the leaking of cells was observed (Arabi et al. 2010).

**Table 1:** Results of 7 days of biotransformation of β-oxoalkylphosphonates to the corresponding diethyl 2-hydroxyphosphonates by free-living and alginate entrapped cells of cyanobacteria, under shaking conditions. Substrates: 1 = diethyl 2-oxopropylphosphonate; 2 = diethyl 2-oxo-2-phenylethylphosphonate; 3 = Diethyl 2-oxobutylphosphonate

Substrate		*N. sphaerocarpa*			*N.* cf. *muscorum*			*A. maxima*		
		Free	1 %	2 %	Free	1 %	2 %	Free	1 %	2 %
1	conversion (%)	25 ± 1	14 ± 3	21 ± 4	-	-	-	16 ± 1	>5	>5
	enantiomeric excess (%)	93 ± 2	93 ± 1	84 ± 5				≥99		
2	conversion (%)	99 ± 0.5	97 ± 1	95 ± 1	43 ± 2	28 ± 1	25 ± 2	–	–	–
	enantiomeric excess (%)	96 ± 0.5	97 ± 0.5	95 ± 1	≥99	≥99	≥99			
3	conversion (%)	27 ± 2	21 ± 3	20 ± 4	–	–	–	–	–	–
	enantiomeric excess (%)	93 ± 0.5	89 ± 4	82 ± 2						

*–* no reaction

Among the tested cyanobacterial strains, the application of entrapped cells of *N. sphaerocarpa* resulted in a slight decrease of the conversion of β-oxophosphonates. *Nodularia sphaerocarpa* cells entrapped in alginate beads were efficient toward compound 2. The degree of conversion and the optical purity of the product diethyl (*S*)-2-hydroxy-2-phenylethylphosphonate were up to 97 % in the case of beads with 1 % (*w*/*v*) alginate concentration. Transformations of aliphatic substrates 1 and 3, by immobilized cells of this strain, were less effective compared to the processes using free-living cells (Table 1); degrees of conversion were 21 and 20 %, respectively, in the case of the use of entrapping beads with 2 % (*w*/*v*) of alginate concentration.

Ca^2+^-alginate-entrapped cells of cyanobacteria exhibit comparable or slightly lower catalytic activity compared to free cells of cyanobacteria, cultured under shaking conditions (Table 1). The use of the agitation, in the case of free cells of cyanobacteria, contributed to the increase of the conversion degree of substrates, since it improves the interaction between the substrate and biocatalyst. This was observed especially in the case of free cells of *N.* cf. *muscorum*, where shaking conditions improved the conversion of diethyl 2-oxo-2-phenylethylphosphonate (2) to 43 % (Table 1), compared to experiments carried out under stationary conditions where the degree of conversion was up to 18 %. In this particular case, the entrapped cells were more efficient biocatalysts than were the free ones cultivated under stationary conditions (Tables 1 and 2). Entrapped cells of *N.* cf. *muscorum* (beads containing 1 and 2 % of final concentration of alginate) were able to convert substrate 2 with similar yield with a conversion degree of 25 and 28 %, respectively, which represents a good result, considering the possibility of reusing the biocatalyst cells.

In the case of bioreduction of aliphatic substrate, diethyl 2-oxobutylphosphonate (3), carried out under shaking conditions with the free cells of *N. sphaerocarpa*, no improvement in the conversion degree was observed, compared to the stationary process. However, agitation improved the optical purity of diethyl 2-hydroxybutylphosphonate (3a)—*e.e.* of this product was up to 93 %. This is indirect proof that the activity of the enzymatic system involved in such bioreduction depends on light availability. Such mechanism was observed in previously described studies.

Immobilized biomass of *A. maxima* exhibited the lowest biocatalytic ability to reduce β-oxophosphonates; the degree of conversion was up to 5 %. In the case of free cells of *A. maxima*, shaking conditions did not influence the effectiveness of the process, compared to the stationary conditions, and the conversion degree was 16 % (99 % of *e*.*e*.).

### Preparative bioreduction of substrate 2 by *Nodularia sphaerocarpa*

Appropriate tests were conducted to evaluate the possibility of the application of free-living and immobilized cells of *N. sphaerocarpa* in preparative scale batch culture and in a continuous-flow photobioreactor. The application of a preparative scale continuous flow bioreactor packed with immobilized cells of *N. sphaerocarpa* achieved 38 % of conversion degree and 86 % of optical purity of the diethyl (*S*)-2-hydroxy-2-phenylethylphosphonate (2a) within the 5-day process. In the case of the 500-mL batch culture of free cells of *N. sphaerocarpa*, the conversion degree was 44 % (91 % of *e.e.* of 2a) with the higher final amount of substrate 2 (5 mM, 540 μL, 640 mg).

**Fig. 1:**
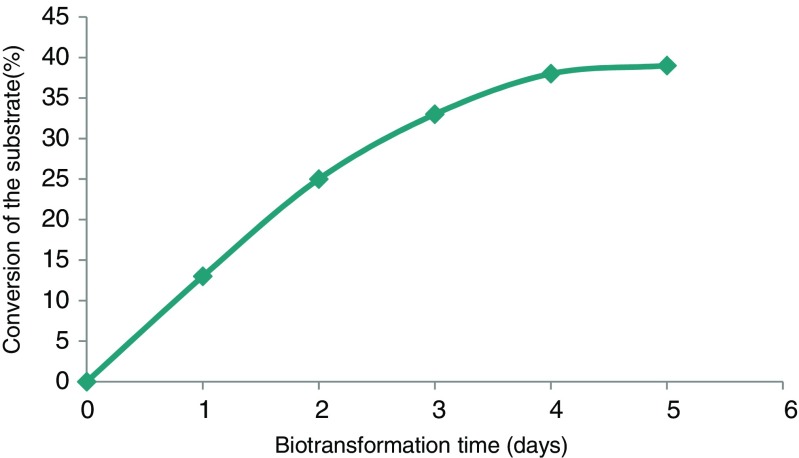
The efficiency of bioreduction of compound 2 during the biotransformation process, in the case of the column bioreactor packing with immobilized cells of *N. sphaerocarpa*

To evaluate the correlation between the reaction progress and the duration of bioconversion, in the case of the column packed bioreactor, appropriated experiments were conducted. Results indicated that the largest increase of conversion degree is observed within the first 48 h of the biotransformation process (Fig. 1).

## Discussion

Previously published results present a new biocatalytic approach to the effective use of photoautotrophic microorganisms as biocatalysts for the synthesis the chiral (*S*)-2-hydroxyphosphonates under stationary conditions (Górak and Żymańczyk-Duda 2015). In contrast to other biological systems, cyanobacteria represent unusual catalytic ability to reduce β-oxoalkylphosphonates and the effectiveness of such reaction strongly depends on the structure of the substrates. Regardless of the conditions used (source of light, photoperiod, cultivation medium), the filamentous cyanobacterium *A. maxima* was a source of reductive activity only toward aliphatic diethyl 2-oxopropylphosphonate (1) (20 % of yield, 99 % of *e.e.*), while *N.* cf. *muscorum* applied as biocatalyst allowed diethyl (*S*)-2-hydroxy-2-phenylethylphosphonate (2a) to be obtained with high enantiomeric excess (99 %) and 26 % of conversion degree (Table 2). *Nodularia sphaerocarpa* was able to reduce the aromatic diethyl 2-oxo-2-phenylethylphosphonate (2); the conversion degree was 99 %, and the optical purity of the product, diethyl (*S*)-2-hydroxy-2-phenylethylphosphonate, was 93 %, under stationary conditions (Table 2). However, previously described results show that the productivity of such a reaction system is affected by substrate concentrations where the degree of conversion of substrate 2 slightly decreases with increasing substrate concentration, but the enantioselectivity of the process and the cell viability remain almost at the same level as confirmed by flow cytometry studies (Górak and Żymańczyk-Duda 2015). These results form the basis for further experiments on scaling-up the processes and to determine if the alteration of the biocatalyst mode allows sustaining the activity of its enzymatic system under particular conditions (higher substrate concentrations). Thus, cyanobacterial cells were entrapped in an alginate matrix, one of the most widely used techniques for immobilization achieved by the physical entrapment of organisms inside a polymeric matrix. This can form a structure with optimized mechanical strength, rigidity, and porosity properties. A major advantage of alginate gel entrapment is that immobilized cells are not exposed to extreme physical–chemical condition changes during the immobilization process, as is observed in the case of immobilization in silica gel (sol–gel technique) (Rangsayatorn et al. 2004). The permeability and low toxicity of the formed matrix creates a gentle environment for the immobilized cells. The concentration of sodium alginate and calcium chloride affects the gelation mechanism and determines the mechanical behavior of alginate gels and their surface morphology (Abdel Hameed 2007). Such a procedure of immobilization seems to be appropriate, because the structure of beads was stable and the elution of cyanobacterial cells from the outer layer of beads was not observed. To evaluate the effect of immobilization on the catalytic activity of biocatalysts, the effectiveness of bioconversion carried out with free-living cells of cyanobacteria (under shaking conditions) and with immobilized cells was compared.

**Table 2:** Results of biotransformation of *β*-oxoalkylphosphonates by free cells of cyanobacteria under stationary (Górak and Żymańczyk-Duda 2015) and shaking conditions. Substrates: 1 = diethyl 2-oxopropylphosphonate; 2 = diethyl 2-oxo-2-phenylethylphosphonate; 3 = Diethyl 2-oxobutylphosphonate

Substrate		*N. sphaerocarpa*		*N.* cf. *muscorum*		*A. maxima*	
		stationary	shaking	stationary	shaking	stationary	shaking
1	conversion (%)	17 ± 2	25 ± 1	–	–	16 ± 3	16 ± 1
	enantiomeric excess (%)	93 ± 2	93 ± 2			≥99	≥99
2	conversion (%)	99 ± 0.3	99 ± 0.5	18 ± 5	43 ± 2	–	–
	enantiomeric excess (%)	93 ± 1	96 ± 0.5	≥99	≥99		
3	conversion (%)	27 ± 3	27 ± 2	–	–	–	–
	enantiomeric excess (%)	80 ± 1	93 ± 0.5				

*–* no reaction

Ca^2+^-alginate-entrapped cells of cyanobacteria transform β-oxoalkylphosphonates to the corresponding (*S*)-hydroxyphosphonates. The presented study demonstrates that alginate-immobilized cells of *N. sphaerocarpa*, *A. maxima*, and *N.* cf. *muscorum* exhibited slightly lower reductive activity toward the β-oxophosphonates in comparison to free cells of the cyanobacteria cultured under shaking condition (Tables 1 and 2). This effect also can be related to the limited amount of light penetrating through the beads which affects the activity of the algal enzymatic system.

As it was shown (Table 1), the influence of the concentration of sodium alginate on the photobiocatalyst activity depends on the cyanobacterial species but the differences in effectiveness are small. Therefore, this cannot be considered as a crucial factor.

A very interesting phenomenon was observed in the case of bioreduction of aliphatic diethyl 2-oxopropylphosphonate (1) by *N. sphaerocarpa*; better results were achieved for cells entrapped in 2 % alginate (21 % of conversion degree (84 % of *e.e.*)) than in the case of using the beads containing ultimately 1 % (*w*/*v*) of alginate (14 % of conversion degree (93 % of *e.e.*)). This is probably associated with the structure of substrate 1, whose moiety is the smallest one and as a consequence, its diffusion across the 2 % alginate membrane is better than in the cases of the other substrates, thus facilitating the biocatalyst–substrate contact. Additionally, the interaction between the converted compound and the biocatalysts is longer in the case of the 2 % matrix compared to the 1 % one, simply because of the diffusion differences as diffusion is easier for beads built with 1 % alginate gel. Considering the other cases, the application of 1 % matrix is more effective than the 2 % one, and this is probably also associated with diffusion differences.

In the case of *N.* cf. *muscorum*, immobilized cells of this species were more efficient than were the free cells cultivated under stationary condition but less effective when shaken culture of free cells of *N.* cf. *muscorum* was used. Thus, the best result was as follows: 43 % of conversion degree and 99 % of optical purity of the (*S*)-2-hydroxy-2-phenylethylphosphonate (2a). This suggests the crucial role of agitation for *N.* cf. *muscorum* application. Agitation allowed the avoidance of photoinhibition and self-shading effect and improves the availability of the substrate to biocatalyst.

Among the tested cyanobacterial strains, the immobilized biomass of *A. maxima* exhibited the lowest biocatalytic ability to reduce β-oxophosphonates within the experimental period (7 days) where the degree of conversion was up to 5 %. This is probably due to the adverse effects of the immobilization on cell viability and catalytic activity as well as on the availability of the substrate to biocatalyst, regardless of the concentration of alginate. That is why the applied procedure of the immobilization of *A. maxima* cells was modified, because the application of *Spirulina* medium as suspension and biotransformation medium resulted in the partial disintegration of structure of supporting matrixes. In the case of media containing EDTA, phosphate, and other cations (e.g., *Spirulina* medium), calcium cations required for proper matrix formation can be sequestered by soluble anions or be substituted by monovalent cations. This results in destabilization of the structure, leading to leakage of algal cells (Hertzberg and Jensen 1989). To avoid this problem, the *Spirulina* medium was replaced by distilled water, such as in the case of the alginate-entrapped cells of *S. platensis* used in biosorption processes (Duda-Chodak et al. 2013). The structure of the matrixes was stable; however, the release of photosynthetic pigments was observed, indicating cell damage. It is possible that the concentration of the cyanobacterial cells used for immobilization purposes was too high—the value of OD_750_ of 21 days of culture of *A. maxima* was 1.6. The initial cell concentration seems to be very important. It was shown that the higher initial cellular density allows a higher final cell density, stable cultures, and more efficient bioprocess to be obtained. On the other hand, too high concentration of cyanobacterial cells entrapped in alginate beads reduce the amount of light penetrating through the beads and enhance the self-shading effects, which limit the metabolic activities of the algal cells. The low catalytic activity of the entrapped cells of *A. maxima* can also result from the deficiency of nutrients observed within the 7-day process of biotransformation. Unfortunately, the application of distilled water instead of *Spirulina* medium resulted in the loss of the cellular activity.

Immobilization procedure and alginate concentration did not affect the results only in the case of use of *N. sphaerocarpa* (except for substrate 1). This, previously described as an effective free-cell biocatalyst able to reduce the β-oxophosphonates (Górak and Żymańczyk-Duda 2015), is the unique species, because of its proven resistance to increasing concentrations of converted xenobiotic substrates. This was a good starting base for further scaling up experiments with the use of *N. sphaerocarpa.*

Previous approaches included experiments with the substrate concentration increasing while maintaining the same volume of the culture of biocatalyst and resulted in decreasing the conversion degree of diethyl 2-oxo-2-phenylethylphosphonate (2) with increasing substrate concentration. Enantioselectivity as well as cell viability remained almost at the same level (Górak and Żymańczyk-Duda 2015). Thus, current efforts involve an increase in both values and were also focused on creating the economic system that allows reuse of biocatalyst.

Biotransformation processes for the best biocatalyst, *N. sphaerocarpa*, on the preparative scale indicated that the use of free-living cells of cyanobacteria is more effective (640 mg of substrate 2, 44 % of yield, 91 % of *e*.*e*.) and provides the suitable reaction condition and viability of biocatalyst compared to the column bioreactor packed with immobilized cells of *N. sphaerocarpa* (384 mg of substrate 2, 38 % of yield, 86 % of *e.e.*)*.* However, such solution with column bioreactor, allows reduction of the volume of required biotransformation medium from 1000 to 150 mL and also allows a significant increase in the substrate concentration to 10 mM (300 μL/ 384 mg), so this is a more economical solution than others (batch culture). However, if the conversion degree, optical purity of the products, and the value of the substrate concentration are considered, the best results were obtained for the process carried out in the 500-mL batch culture of free cells of *N. sphaerocarpa*.

An important observation is that the results obtained for the 500-mL batch culture of free cells of *N. sphaerocarpa* are in the range of 35–59 % of conversion degree and are comparable to those which were obtained in the case of use the 6 and 4 mM concentration of substrate 2, for laboratory-scale preparations (100 mL) (Górak and Żymańczyk-Duda 2015). We conclude that the applied physical–chemical conditions of batch culture are close to the optimal ones, which were worked out in the laboratory-scale experiments.

In the case of the column packed bioreactor, the correlation between the reaction progress and the duration of bioconversion shows that with over time, the efficiency of bioreduction decreased (Fig. 1) and the largest increase of conversion degree is reached within the first 48 h. Such a phenomenon is a consequence of the self-shading effects in the column packed with immobilized cells resulting in the limitation access to the light, which seems to be crucial for the biocatalytic activity of *N. sphaerocarpa* cells. However, the viability of the cells remains almost at the same level, which in turn is the next indirect evidence that enzymatic systems involved in the phosphonate conversion are a part of secondary metabolic pathways and are not vital for the survival of microbial cells.

This study for the first time reports successful scaling up the whole-cell biocatalyzed conversion of prochiral oxophosphonates. What is also important is that this study showed that there is the possibility to reuse the immobilized cells of a photobiocatalyst. Thus, the application of two different approaches, 500-mL batch culture and continuous column bioreactor, resulted in obtaining the desired, chiral product (*S*)-2-hydroxy-2-phenylethylphosphonate (2a) with good yield and satisfactory optical purity. The cells of *N. sphaerocarpa* remain metabolically active after completing the reduction of diethyl 2-oxo-2-phenylethylphosphonate (2). This is a good starting point for its further applications.
